# The role of partners in promoting self-care for misoprostol and subcutaneous DMPA in Pakistan

**DOI:** 10.1186/s12961-021-00714-0

**Published:** 2021-04-21

**Authors:** Qudsia Uzma, Nausheen Hamid, Rizwana Chaudhri, Nadeem Mehmood, Atiya Aabroo, Ellen Thom, Karima Gholbzouri, Ramez Mahaini, Nilmini Hemachandra

**Affiliations:** 1 Maternal, Newborn, Child, Adolescent Health, WHO Country Office, Islamabad, Pakistan; 2 Ministry of National Health Services, Regulations and Coordination, Government of Pakistan, Islamabad, Pakistan; 3 Rawalpindi Medical University, Rawalpindi, Pakistan; 4 Rahnuma–Family Planning Association of Pakistan, Lahore, Pakistan; 5 Ministry of National Health Services, Regulation & Coordination, Government of Pakistan, Islamabad, Pakistan; 6 Lead for Promoting Health Through Life Course, WHO Country Office, Islamabad, Pakistan; 7 Reproductive and Maternal Health, Department of Health Promotion, WHO Regional Office for the Eastern Mediterranean, Cairo, Egypt; 8 Department of Health Promotion, WHO Regional Office for the Eastern Mediterranean, Cairo, Egypt; 9 Sexual and Reproductive Health and Rights,, WHO Regional Office for the Eastern Mediterranean, Cairo, Egypt

## Abstract

**Background:**

Pakistan is among a number of countries facing protracted challenges in addressing maternal mortality with a concomitant weak healthcare system complexed with inequities. Sexual and reproductive health and rights (SRHR) self-care interventions offer the best solution for improving access to quality healthcare services with efficiency and economy. This manuscript documents country experience in introducing and scaling up two selected SRHR self-care interventions. A prospective qualitative study design was used and a semi-structured questionnaire was shared with identified SRHR private sector partners selected through convenience and purposive sampling. The two interventions include the use of misoprostol for postpartum hemorrhage and the use of subcutaneous depomedroxyprogesterone acetate (DMPA) as injectable contraceptive method. Data collection was done through emails and telephone follow-up calls.

**Results:**

Nine of the 13 partners consulted for the study responded. The two selected self-care interventions are mainly supported by private sector partners (national and international nongovernmental organizations) having national or subnational existence. Their mandates include all relevant areas, such as policy advocacy, field implementation, trainings, supervision and monitoring. A majority of partners reported experience related to the use of misoprostol; it was introduced more than a decade ago, is registered and is procured by both public and private sectors. Subcutaneous DMPA is a new intervention, having been introduced only recently, and commodity availability remains a challenge. It is being delivered through health workers/providers and is not promoted as a self-administered contraceptive. Community engagement and awareness raising is reported as an essential element of successful field implementation; however, no beneficiary data was collected for the study. Training approaches differ considerably, are standalone or integrated with SRHR topics and their duration varies between 1 and 5 days, covering a range of cadres.

**Conclusion:**

Pubic sector ownership and patronage is essential for introducing and scaling up self-care interventions as a measure to support the healthcare system in delivering quality sexual and reproductive health services. Supervision, monitoring and reporting are areas requiring further support, as well as the leadership and governance role of the public sector. Standardization of trainings, community awareness, supervision, monitoring and reporting are required together with integration of self-care in routine capacity building activities (pre- and in-service) on sexual and reproductive health in the country.

## Background

The results of the recently conducted demographic and health survey in Pakistan reflected that some progress has been made on key maternal and child health indicators in Pakistan [1]. Antenatal care—at least one visit (86%), facility-based deliveries (66%) and neonatal mortality (42/1000 live births) are among the areas showing reasonable progress. However, the reproductive health/family planning indicators reflect that progress is very slow, with a stagnant—actually, declining—modern contraceptive prevalence rate of 26%, as reflected in the trend of total fertility rate (overall: 3.6; 2.9 in urban areas and 3.9 in rural areas) [1]. Since the last census in 1998, the population has grown by 57% in 20 years, and the crude birth rate, though gradually declining, is still alarmingly high at 22 births per 1000 people [1].

Pakistan is facing the serious challenge of reducing its high maternal mortality ratio (140/100 000 live births) [2], requiring a much faster annual rate of reduction in order to achieve the target (70/100 000 live births) set in Sustainable Development Goals 2030 [2]. A pregnant woman’s chance of survival drops considerably due to limited access to antenatal care (ANC) ≥ 4 visits (36.6% in 2012–2013 and 51.4% in 2017–2018 [3]), restricted medical supplies, poor healthcare availability and lack of health infrastructure. Preventable maternal morbidity and mortality remains an intimidating and failed public health challenge. Haemorrhage, persistently the leading cause of maternal deaths [4], is a preventable event in the majority of the cases, especially through interventions like the use of oxytocic drugs, such as misoprostol.

Evidence shows that the proportion of pregnancies that are unintended is on an increasing trend, with a high adolescent birth rate (38/1000 girls aged 15–19 years) and an even higher unmet need for family planning (17%). More than half of these unintended pregnancies result in induced abortions, and complications related to unsafe abortion is one of the five leading causes of maternal deaths in the country.

The country’s baseline for the Universal Health Coverage (UHC) index is reported to be very low, 40 [5], indicative of poor access and use of essential health services, and is even worse than that of sub-Saharan Africa [5]. The UHC index is reported on a unitless scale of 0 to 100 and is computed as the geometric mean of 14 tracer indicators of health service coverage. The tracer indicators are organized by four components of service coverage: (1) reproductive, maternal, newborn and child health; (2) infectious diseases; (3) noncommunicable diseases; (4) service capacity and access [6]. Within the reproductive, maternal, newborn and child health component, the main indicators include prevalence rates of modern contraceptive methods and antenatal care ≥ 4 visits and two child health indicators. Therefore, country performance on sexual and reproductive health issues has a direct effect on its UHC index.

The main reason for such a poor performance is the low coverage of sexual and reproductive health and rights (SRHR) essential services, with serious equity issues and low healthcare expenditure. In such a situation, where health system is facing chronic challenges in meeting the healthcare needs of women and couple and ensuring equitable coverage and good quality of SRHR services, it is critically important to promote use of evidence-based self-care for SRHR interventions benefitting the entire population. WHO’s definition of self-care is the ability of individuals, families and communities to promote health, prevent disease, maintain health and cope with illness and disability with or without the support of a health worker.

Self-care interventions recommended by WHO are evidence-based and can include information about a sexual or reproductive health issue as well as ways in which individuals can obtain drugs, devices, diagnostics and /or digital products fully or partially separate from formal health services that can be used with or without the direct supervision of a health worker.

SRHR self-care is being practiced by women for millennia, including management of menstruation, contraception, pregnancy and childbirth. Over time, self-care has become sophisticated and data-driven. Evidence shows that when people are active participants in their own healthcare, adherence to treatment regimens improves [7]. In the long run, self-care is of economic interest from both a community and a personal perspective, as it effects a relief for the healthcare system and results in changed healthcare seeking behaviours [8]. Self-care can increase the level of engagement and autonomy that people can exercise over their health, providing the opportunity to improve equitable access to healthcare, quality of care and financial protection for the users of self-care [9]. Self-care brings benefits, such as improving the efficiency of healthcare delivery, by including users as lay health workers, increasing the use of preventive services and adoption of preventive behaviors, improving adherence to treatments and reducing the need for the formal healthcare services [10].

With the current COVID-19 situation, self-care has become even more relevant and essential. In order to address the sexual and reproductive health statusand ensure equitable access to SRHR care services, partners are reporting that they have initiated efforts for community sensitization on key SRHR self-care interventions in line with WHO global recommendations. In this context, a rapid exercise to landscape current experiences on two selected SRHR interventions was conducted through online communication with relevant partners in Pakistan. The selected SRHR interventions were: (1) interventions that included the use of misoprostol for the prevention and management of postpartum hemorrhage (PPH); (2) the use of subcutaneous (SC) depomedroxyprogesterone acetate (DMPA) for contraception. The key overall objective of this study was to document the implementation of self-care for SRHR interventions experiences in the Pakistan context and identify major challenges for informing future planning and scale-up. This is the first time such a study has been conducted in Pakistan and the results will contribute to the existing literature.

The aim of this study is to document the best practices and lessons learnt from introducing and implementing the use of misoprostol and SC DMPA as self-care SRHR interventions. The specific objectives of the study are:

1. To explore the role of partners in planning for the implementation of two identified SRHR self-care interventions in Pakistan
2. To determine the implementation processes used by partners for introducing and delivering the two identified SRHR self-care interventions in Pakistan.

## Methods

This was a prospective qualitative study involving nongovernmental organizations (NGOs) in the country as partners that have had any experience implementing the selected SRHR self-care interventions. The study team, including representatives from the Ministry of National Health Services, Regulations and Coordination (NHSR&C), Government of Pakistan, WHO Eastern Mediterranean Regional Office (EMRO) and Country Office and a partner organization, reviewed a detailed list of partner organizations involved in sexual and reproductive health programmes in the country to identify potential partners having experience in self-care SRHR interventions. A convenience sample of partners was derived for seeking inputs on a structured questionnaire.

A simple structured questionnaire was developed to capture key highlights of each partner’s experiences in introducing/implementing the two SRHR self-care interventions in the country. The questionnaire included sections on basic information on the organization, such as type of organization, its geographic coverage in the country and technical areas of focus related to SRHR programming. There are specific questions regarding the partner’s involvement in capacity-building activities, duration of trainings, approach of training (i.e. as stand-alone or integrated with other SRHR topics), availability of master trainers and estimated number and type of providers trained. Other questions related to the partner’s role in community outreach, monitoring and supervision, data management and reporting, as well as on specific contributions during COVID-19 response. The inputs of each partner on the challenges and lessons learnt were also sought through open-ended questions. The questionnaire did not include any data on beneficiaries, and no community interaction was included in the methodology.

A list of partners was drafted in consultation with relevant experts in the SRHR field, and the questionnaire was circulated by the focal point at the Ministry of NHSR&C to all of the listed partners. The questionnaire was shared with a total of 13 partners, of which two responded that they had no relevant experience. Responses were received from 70% of partners (*n* = 9), and 30% did not respond despite two reminders by emails. The feedback received was collated and findings were documented accordingly.

**Fig. 1:**
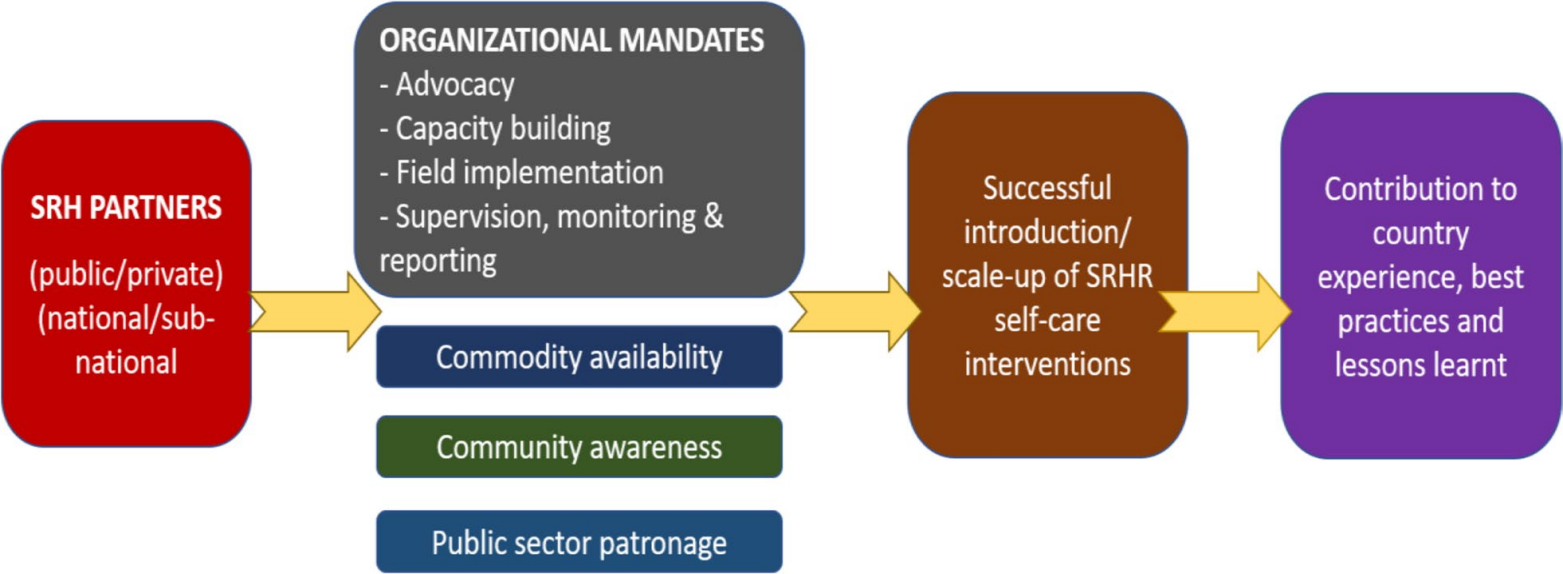
Conceptual framework for exploring partner’s contribution to sexual and reproductive health and rights (*SRHR*) self-care experiences

A conceptual framework (Fig. 1) was developed to guide the process of documenting country experience in SRHR self-care interventions—use of misoprostol and SC DMPA.

## Results

### Organization profile and health coverage through self-care for SRHR interventions

The findings show that key perpetrators of this important intervention are mainly private sector, namely civil society organizations working on SRHR in the country. Of the total of nine respondents, five belonged to international NGOs and four were from local NGOs. Key actors involved in SRHR self-care implementation in the country are IPAS, Jhpiego, International Medical Corps, Greenstar Social Marketing, National Committee on Maternal and Newborn Health, AMAAN, Rahnuma–Family Planning Association of Pakistan, Pathfinder International and Riz consulting, among others. More than half of the partners involved in the introduction/implementation of SRHR self-care interventions have geographic coverage across all four provinces of Pakistan with five of the nine respondents having subnational presence and the remaining four working on national scale. Almost all partners have had a role in policy advocacy for SRHR, technical support in guidelines/training modules development, capacity building and follow-up, implementation on the ground as well as supervision and monitoring (Table 1).

**Table 1:** Organizational profiles related to sexual and reproductive health and rights programmes

Respondent number	Experience with misoprostol or SC DMPA?	Organization’s profile related to SRHR programming in Pakistan						
		Policy advocacy for SRHR services	Technical support for guidelines/ training modules/ reporting tools etc	Training of trainers	Cascade training	Training follow ups	Implementation in field	Supervision and monitoring
1	Misoprostol	√	√	√	√	√	√	√
2	SC DMPA	√	√	√	√	√	√	√
3	Misoprostol	√					√	√
4	Misoprostol	√		√	√	√	√	√
5	Misoprostol	√	√	√	√	√	√	√
6	Misoprostol	√	√	√	√		√	√
7	Both	√	√	√	√	√	√	√
8	Both	√	√	√	√	√	√	√
9	SC DMPA	√		√				

*DMPA* Depomedroxyprogesterone acetate,* SC* subcutaneous,* SRHR* sexual and reproductive health and rights

### Implementation process

More than half of the partners were not informed about global WHO guidelines on SRHR self-care interventions while they initiated the introduction/implementation of these activities. The majority of partners have experience with misoprostol but few of them have experience with SC DMPA alone or with both interventions.

#### Policy advocacy

All except one of the partners are involved in policy advocacy on sexual and reproductive health in Pakistan, including a focus on either or both of the selected self-care interventions. Policy advocacy is found as a mandate adopted by both national and international NGOs. Misoprostol is an example of the effectiveness of this role as it was first recommended as the medicine of choice for the treatment of PPH and post-abortion care under the auspices of the Maternal, Newborn, and Child Health (MNCH) Best Practices Programme launched in 2009. Subsequently, it was registered with the Drug Regulatory Authority of Pakistan and was included in the essential medicine list. It is being regularly procured by the public and private sector, is also available over-the-counter at pharmacies at an affordable price and is widely used as a routine care intervention by skilled birth attendants as well as a self-care intervention.

#### Capacity building activities

**Fig. 2:**
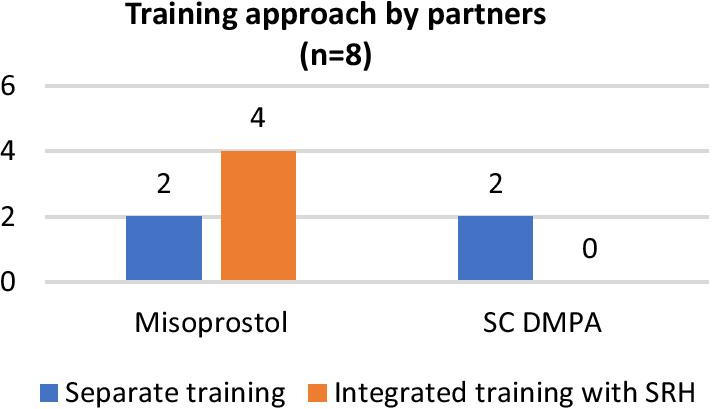
Training approach adopted by SRHR partners who shared their self-care experience

**Fig. 3:**
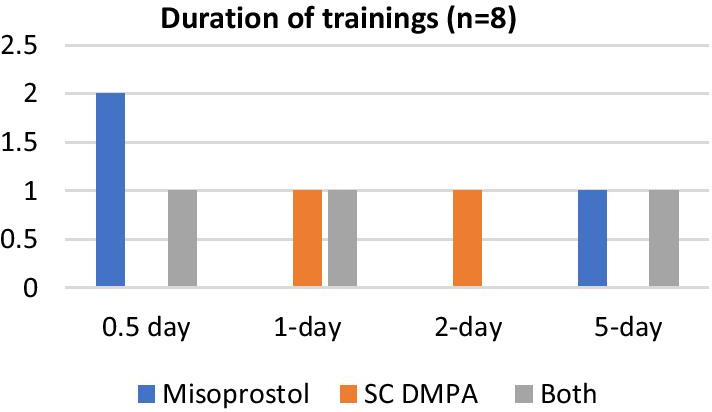
Duration of training sessions on selected two SRHR self-care interventions. *SC DMPA* Subcutaneous depomedroxyprogesterone acetate

All partners have had capacity building interventions involving various cadres of health providers and communities for the use of misoprostol while focussing on medical doctors, including midlevel providers for SC DMPA. These trainings were conducted either as separate sessions or integrated with SRHR trainings (Fig. 2). The duration of trainings has been reported as variable, ranging between 0.5 and 5 days depending on the type of participant (Fig. 3). For example, pharmacists were oriented in 0.5 day and data collectors were trained in 5 days, while pregnant women were sensitized in 0.5 day, managers were trained in 2 days and health providers were trained in 1 day.

Partners have varied experience in imparting trainings, with some having conducted as many as 400 training sessions while others have only conducted one session (Table 2). Training sessions on the use of misoprostol outnumber those on SC DMPA or both, as experienced by some of the partners. The number of providers/managers trained on SRHR self-care interventions range from 30 to more than 2000 by different partners (Table 2).

**Table 2:** Number of trainings and providers trained by partners on self-care SRHR interventions

Respondent number	Conducted trainings on self-care? (yes/no)	Estimated number of training sessions to date	Estimated number of providers trained	Duration of training	Training implemented as:	
					Separate session	Integrated with SRHR
1	Yes	17	1980	5 days	√	√
2	Yes	> 40	666	1 day	√	–
3	No	–	–	–	–	–
4	Yes	> 60	1900	30 min to 0.5 day	–	√
5	Yes	2	50	0.5 day	–	√
6	Yes	> 400	> 1000	0.5–1 day	√	√
7	Yes	8	75	1 day	–	√
8	Yes	116	> 100	5–6 days	–	√
9	No	1	30	2 days	√	–

The cadres trained on these self-care interventions include doctors, nurses, midwives, lady health visitors, lady health workers and counsellors. Project staff, health managers and community volunteers have been trained on the use of misoprostol but not on SC DMPA. None of the partners reported training cadres working under the auspices of population welfare departments (family welfare workers) on use of misoprostol although they are included in trainings on SC DMPA (Table 3). All partners were in agreement that self-care SRHR interventions present a promising strategy during the current COVID-19 situation for supporting continuation of essential SRHR services.

**Table 3:** Types of cadres trained by SRHR partners who shared their self-care experience

Respondent number	Trained on both self-care interventions (misoprostol and SC DMPA)					Trained on use of SC DMPA	Trained on use of misoprostol		
	Medical doctors	Nurses	Midwives	Lady health visitors	Counsellors	Family welfare workers	Project staff	Community volunteers	Programme managers
1	√	√	√	√	–	√	√	–	√
2	√	√	√	√	√	√	–	–	√
3	–	–	–	–	–	–	–	–	–
4	√	√	√	√	√	√	–	–	√
5	√	√	√	√	√	√	–	–	–
6	√	√	√	√	√	√	√	√	–
7	–	√	√	√	–	–	–	–	√
8	√	–	√	√	–	√	√	–	√
9	√	–	√	√	√	–	–	–	–

#### Field implementation

Partners involved in SRHR programmes in the country supported the introduction/implementation of SRHR self-care interventions. With their capacities in policy advocacy, imparting trainings, reporting, supervision and monitoring, the partners reported to have been able to address implementation challenges. In order to promote self-use of misoprostol, partners used different strategies. One approach was to distribute “Safe Delivery Kits” that contained three tablets of misoprostol (with instructions in the local language for normal delivery, newborn care and PPH prevention with misoprostol) in addition to a dignity sheet, apron, soap, cord clamp among other items. These kits were given to all pregnant women in camps and even hospitals wards with the goal to ensure that active management of third-stage of labor (AMTSL) took place. The women were instructed that they must take these three tablets (oral/sublingual) after the baby was born. Other strategies adopted by the partners include the use of helplines to address frequently asked questions and community follow-ups. Field implementation experiences show that even rural women with low literacy welcome learning on SRHR self-care; they follow instructions and take responsibility provided they receive clear guidance. Effective counselling with users can support the continuity of SRHR self-care and greatly reduce the need for referral of PPH cases to hospitals (in the case of misoprostol). Experience from the field implementation of these interventions indicate that user-friendly items (like SC-DPMA) can promote the uptake of contraception but that the government’s role to ensure enabling environment and commodity security is highly essential.

#### Supervision and monitoring

For monitoring and supervision, most of the partners have reported using a systematic process including on-the-job mentoring, refreshers, follow-ups, field supervisory visits and regular reporting strategies. The majority of partners are not sharing the relevant data with government counterparts as a routine. Information sharing is done through dissemination events by some partners.

#### Commodity availability

In terms of the commodity availability, it is important to note that misoprostol is registered in the country and is included in the essential medicine list for management of PPH. This was made possible through sustained advocacy by partners which ultimately resulted in its registration by the Drug Regulatory Authority of Pakistan, following which it is being procured widely by both public and private sectors and is readily available on the market. On the contrary, SC DMPA is a newly introduced contraceptive method and is being imported by organizations involved in its introduction and implementation. It is yet to be registered and included in the essential list of contraceptive methods. Partners report the procurement of these commodities by their organization or by another organization and supplied to their organization (including donations from a pharmaceutical company). Regarding misoprostol, clients may also need to make an out-of-pocket payment in situations where it is not made available free of cost by the provider. In the case of SRHR self-care interventions, there is a lot of reliance on over-the-counter availability of commodities and supplies; therefore, it is of utmost importance to ensure capacity building and on-boarding of chemists/pharmacists to facilitate the introduction/implementation of SRHR self-care interventions in the country.

#### Community awareness

The partners who reported being informed of the global WHO guidelines on SRHR self-care interventions also indicated that they played a key role in enhancing community awareness on the use of identified self-care SRHR interventions. This included orientation sessions with pregnant women, counsellors, lady health workers and community volunteers as well as with informal providers such as pharmacists, who are able to contribute to the community awareness.

#### Public sector patronage

For misoprostol, ownership by the public sector in particular for its procurement and distribution through the public sector healthcare delivery system has been pivotal in its wide-scale implementation. Similarly, SC DMPA is also introduced in consultation with the public sector mainly with consent of the Population Welfare Department in Sindh province where it was piloted for the first time in 2018. For both interventions, all capacity building activities involved mainly the public sector providers and managers in addition to some project staff, volunteers and community members.

### Self-care interventions during COVID-19 response

All partners were in agreement that the response to COVID-19 has provided opportunities for expanding the use of self-care SRHR interventions. Only two of the nine respondents reported having no active role during the COVID-19 response in community outreach programmes with a focus on SRHR self-care interventions. Among the partners who responded positively, various alternative strategies were reported for reaching out to the communities remotely. These include setting up a SHRH telehealth line (toll free) linking communities with expert clinicians to provide SRHR consultations and the use of E-messages and telehealth, alongside the use of conventional approaches, such as community educators, 24/7 helpline etc. However, commodity security remains a key concern for all and limits the expansion of such alternate strategies to benefit the larger population.

## Discussion and conclusion

The use of misoprostol as SRHR self-care intervention in Pakistan has been implemented for more than a decade, mainly perpetuated through the private sector. It was introduced much before the recommendation by WHO as a self-care intervention, and most of the partners are not aware even now of the global WHO guidelines on SRHR self-care interventions. SC DMPA, however, is a newer contraceptive method and has been introduced only recently with support from private sector partners. Within Pakistan, there has been little integration of self-care interventions into the public sector capacity building or service delivery mechanisms.

In line with the conceptual framework for the WHO consolidated guideline on self-care interventions [11], findings from this study reveal that a “people centred” approach remains the core objective of promoting self-care interventions with the objective of empowering communities. Similarly, the enabling environment for such introduction and field implementation requires “health systems” components, such as service delivery, essential medicine, human resources and information systems etc. In the same context, the places of access and roles of pharmacies or digital platforms as well as of the communities themselves are closely linked with the introduction, uptake and scale-up of self-care interventions.

*Policy advocacy* on sexual and reproductive health is part of the organizational mandate of almost all partners involved in the SRHR self-care interventions, providing these partners with the opportunity to contribute to the creation of an enabling environment through sustained advocacy on promoting SRHR self-care interventions in the country. A relevant example is that of misoprostol. With sustained advocacy of public health practitioners, misoprostol was included in the list of essential medicines for management of PPH after the launch of MNCH Best Practices in 2009 (Karachi Declaration 2009) [12].

*Field implementation* is another mandate shared by many partners who responded to this study. Despite the fact that the majority of responders were not aware of the WHO recommended SRHR self-care interventions, they played a critical role in the introduction and scale-up of these interventions in the country. The key factors driving this interest and motivation of the partners to promote these interventions were the scientific evidence underlying the use of the medicine (misoprostol and SC DMPA). Other reasons include the greater need for engaging women and communities during crises, such as the floods in 2010, and the poor situation of family planning indicators, as reported in the Pakistan Demographic and Health Survey 2018, emphasizing the dire need to address the uptake of contraceptive methods, preferably by self-administration. It is pertinent to note that despite the WHO recommendation on self-administration of SC DMPA, in Pakistan, the introduction phase is involving community-based workers (lady health workers) for providing the family planning commodity with the plan for scaling-up through women’s self-administration in the future. Research across several countries has demonstrated that most women are able to self-inject SC DMPA provided they have been offered the proper training [13]. Therefore, once the self-administration of SC DMPA is officially allowed in the country, the implementation approach can be easily modified to involve women for self-use rather than relying on the health workers.

*Capacity building* of human resources is an important element of successful implementation. A good practice statement in the WHO guidelines on self-care highlights the importance of trained human resource:

“Health-care workers should receive appropriate recurrent training and sensitization to ensure that they have the skills, knowledge and understanding to provide services.” [14]

Almost all partners (except one) have reported making significant contributions in terms of capacity building of the health workforce, including various cadres such as doctors, nurse, midwives, counsellors, community-based workers and volunteers along with managers. It is pertinent to note that trainings on misoprostol rarely include cadres from population welfare departments (i.e. family welfare workers) whose prime focus is on family planning services and are not considered to be skilled birth attendants. On the contrary, the same cadre is among the others who receive trainings on the use of SC DMPA since they are all expected to deliver family planning services. However, the training approaches being used by partners are quite varied. The topics are either given separately or integrated with the SRHR trainings, with the total duration of the training sessions ranging between half a day to 5 days, as reported by partners. These varied approaches indicate the need to have standardized training material for ensuring the quality of trainings and also avoiding any conflicting messages being forwarded to the communities and women due to the lack of a standardized training content. The Global Values and Preferences Survey (GVPS) found that many healthcare providers wanted more training or information about these self-care interventions and were also concerned about patients not using the intervention correctly and not accessing healthcare if/when needed. WHO guidelines emphasize that for the promotion and facilitation of self-care interventions for SRHR, it is important that training for health workers incorporates: communication to enable informed decision-making; values clarification; interprofessional teamworking; and empathetic and compassionate approaches to care. [13] It is therefore essential for sustained scale-up in Pakistan to have standardized training approaches with a focus on the key elements as per the global guidance. An equally important aspect is the training of users on self-administration, self-monitoring and self-awareness. The WHO guidelines recommend that healthcare providers in low-resource settings may find the option to task-shift the administration of injectable contraception to clients themselves acceptable, as long as effective training can be provided and the safety of users can be assured. However, while doing so, it must be ensured that the users/clients are given the option to still seek services from healthcare providers for such interventions, if they prefer so or are hesitant or concerned about doing so themselves.

Based on experience reported in Pakistan, the training of communities, especially women, has been focused on by some partners, and this needs to be applied across the board as a pre-requisite for introducing and scaling up self-care SRHR interventions.

Although self-care interventions are recommended by WHO for adoption by communities in the larger interest of UHC and being cost-effective for the end-user, higher initial investment by the health system is required for the provision of training, supervision and monitoring [10]. However, for cervical cancer screening, the recommended self-care intervention is found to be associated with increased uptake (women twice likely to self-use) regardless of supervision. In experience gained from Pakistan, the use of misoprostol has been widespread without much documented supervision by partners and the public sector healthcare system. On the other hand, SC DMPA is not yet allowed as a self-administered contraceptive and is being delivered through health providers and community-based health workers to ensure appropriate usage and reporting. Likewise, for the health workforce involved in promoting and implementing user-led approaches and autonomy through self-care interventions, pre- and in-service training and on-the-job supervision are equally important. Moreover, a fair number of recommendations by WHO on self-care interventions require an assurance of targeted monitoring and follow-up or referrals to ensure that the users are well supported by the healthcare system. Self-monitoring by users brings benefits, such as reduced frequency of hospital visits and increased uptake of self-care medical devices or commodities [14]. Monitoring and oversight are also important to ensure that standards are implemented and maintained. It is important to provide mechanisms for anonymous reporting to anyone who may experience stigma and/or discrimination when they try to obtain health services. [10]

*Commodity security* is another critical element for ensuring the scale-up of self-care intervention. Subsequent to its registration and inclusion in the essential medicine list in Pakistan, misoprostol is now routinely procured by the governmental departments of health; it is easily available at an affordable price and is being used widely at healthcare facilities in both the public and private sector. This availability has also allowed the use of misoprostol for medical abortion, as per the global guidelines and also practiced in the country, although the medicine is only registered for PPH. Almost all partners have reported issues on the availability of SC DMPA as all supplies have been imported for the sake of operational research, and early introduction was managed by the United Nations Population Fund in 2018 and 2019. New and improved products and tools may help women exercise autonomy over their sexual and reproductive health using these self-care interventions; however, continuous uninterrupted availability of commodities and tools remain a challenge worldwide. Professional groups, such as the Oral Contraceptives Over-the-Counter Working Group, which maps global availability, have long been advocating that women need to be given the control of their SRHR care by allowing non-prescription access to oral contraceptives [13]. The same would hold true for other commodities and tools as per the recommended self-care interventions, including misoprostol and SC DMPA.

*Community awareness*, especially the orientation of women, about the self-care interventions needs to be further enhanced by all partners as well as the players in public sector. Just as trainings are necessary for the health workforce, women would also require support and orientation on the use of self-care interventions—at times with back-up from the relevant health workforce. Encouraging, preparing and supporting women and girls to take ownership of certain aspects of their SRHR care can lead to multiple benefits for both the health system and the users. These include women learning about their bodies and having increased self-sufficiency and health workers freed up to devote more time for other medical conditions.

## Study limitations

This paper used a prospective qualitative study design to investigate self-care implementation experiences using a feasible sample of selected partners (mainly NGOs) [14]. Emails and telephone communication were used with a pre-structured questionnaire as a relevant data collection technique [15]. The validity and reliability are also considered to be sufficient as the questions were constructed by a group of public health experts and were adequately responded to by the partners. Only a few partners declined, and this only because they did not have sufficient implementation experience to report. There were no questions included to capture details on community engagement and beneficiary data.

## Data Availability

All data generated or analysed during this study are included in this published article and supplementary information files have also been uploaded.

## References

[1] National Institute of Population Studies. Pakistan Demographic and Health Survey. Islamabad: National Institute of Population Studies, 2018–19.

[2] WHO, UNICEF, UNFPA, World Bank Group and the United Nations Population Division. Maternal mortality in 2000–2017.https://www.who.int/gho/maternal_health/countries/pak.pdf?ua=1. Accessed 15 Jul 2020.

[3] UNICEF, WHO, World Bank, UN-DESA Population Division. Levels and trends in child mortality. 2015. https://www.who.int/maternal_child_adolescent/documents/levels_trends_child_mortality_2015/en/. Accessed 18 Jul 2021

[4] National Institute of Population Studies. Pakistan Demographic and Health Survey. Islamabad: National Institute of Population Studies, 2006–2007.

[5] Khalid F, Brunal MP, Sattar A, Laokri S, Jowett M, Raza W (2020). Assessing the efficiency of sub-national units in making progress towards universal health coverage: evidence from Pakistan. Health Syst Reform..

[6] WHO. UHC service coverage index. https://www.who.int/data/gho/indicator-metadata-registry/imr-details/4834#:~:text=Target%203.8%20is%20defined%20as,medicines%20and%20vaccines%20for%20all%E2%80%9D. Accessed 17 Jul 2020.

[7] Levinson W, Lesser CS, Epstein RM (2010). Developing physicians communication skills for patient-centered care. Health Aff.

[8] Marklund B, Almroth B, Schaffrath AM, Gunnarsson B, Höijer B, Fridlund B. Promoting medical self-care: evaluation of a family intervention implemented in the primary health care by pharmacies. Fam Pract. 1999;16(5):522–7. 10.1093/fampra/16.5.522. 10.1093/fampra/16.5.522 10533951

[9] Remme M, Narasimhan M, Wilson D, Ali M, Vijayasingham L, Ghani F (2019). Self care interventions for sexual and reproductive health and rights: costs, benefits, and financing. BMJ.

[10] Panagioti M, Richardson G, Small N (2014). Self-management support interventions to reduce health care utilisation without compromising outcomes: a systematic review and meta-analysis. BMC Health Serv Res.

[11] World Health Organization (2019). WHO consolidated guideline on self-care interventions for health: sexual and reproductive health and rights.

[12] Ministry of Health & Ministry of Population Welfare, Government of Pakistan. Karachi Declaration: Scaling up Maternal, Newborn, Child Heath and Family Planning Best Practices in Pakistan. https://phkh.nhsrc.pk/sites/default/files/feeds/Karachi%20Declaration%20MNCH%20and%20FP%20Best%20Practices%20Pakistan%202009.pdf. Accessed 20 Jul 2020.

[13] https://path.azureedge.net/media/documents/RH_Outlook_Nov_2017.pdf. Accessed 20 Jul 2020.

[14] Burns N, Grove SK (1993). The practice of nursing research; conduct, critique and utilisation.

[15] Rapley P (1997). Self-care: re-thinking the role of compliance. Aus J Adv Nurs.

